# The effect of Lu AG09222 on PACAP38- and VIP-induced vasodilation, heart rate increase, and headache in healthy subjects: an interventional, randomized, double-blind, parallel-group, placebo-controlled study

**DOI:** 10.1186/s10194-023-01599-w

**Published:** 2023-05-25

**Authors:** Nadja Bredo Rasmussen, Christina Deligianni, Casper Emil Christensen, William Kristian Karlsson, Haidar Muhsen Al-Khazali, Tom Van de Casteele, Charlotte Granhall, Faisal Mohammad Amin, Messoud Ashina

**Affiliations:** 1 Department of Neurology, Danish Headache Center, Rigshospitalet Glostrup, Faculty of Health and Medical Sciences, University of Copenhagen, Valdemar Hansen Vej 5, DK-2600 Glostrup, Denmark; 2 Department of Clinical Medicine, Faculty of Health and Medical Sciences, University of Copenhagen, Valdemar Hansen Vej 5, 2600 Glostrup, Denmark; 3 H. Lundbeck A/S, Ottiliavej 9, 2500 Valby, Denmark; 4 Department of Neurorehabilitation/Traumatic Brain Injury, Rigshospitalet Glostrup Faculty of Health and Medical Sciences, University of Copenhagen, Valdemar Hansen Vej 5, DK-2600 Glostrup, Denmark

**Keywords:** Migraine, Pituitary adenylate cyclase-activating polypeptide, Migraine disorders, Monoclonal antibody, Migraine treatment

## Abstract

**Background:**

Pituitary adenylate cyclase-activating polypeptide (PACAP), structurally related to vasoactive intestinal peptide (VIP), is one of the important mediators in the pathogenesis of migraine and is known to dilate cranial arteries and induce headache and migraine. Our objective was to determine whether Lu AG09222—an investigational humanized monoclonal antibody directed against PACAP ligand—would inhibit the PACAP-signaling cascade by abolishing its vasodilatory and headache-inducing abilities.

**Methods:**

In a randomized, double-blind, parallel-group, single-dose, placebo-controlled study of Lu AG09222, healthy volunteers aged 18–45 years without history of headache disorders were randomly allocated to three treatment sequences (1:2:2) on two experimental infusion visits with 9 ± 3 days’ interval: placebo + saline + saline (*n* = 5), placebo + PACAP38 + VIP (*n* = 10), and Lu AG09222 + PACAP38 + VIP (*n* = 10). The primary outcome measure was area under the curve (AUC) of the change in superficial temporal artery (STA) diameter from 0 to 120 min after start of infusion of PACAP38. The study was conducted at the Danish Headache Center in Copenhagen, Denmark.

**Results:**

In participants who received Lu AG09222 + PACAP38 infusion, there was a significantly lower STA diameter (mean (SE) [95% CI] AUC ‒35.4 (4.32) [‒44.6, ‒26.3] mm × min; *P* < 0.0001) compared to participants who received placebo + PACAP38 infusion. Secondary and explorative analysis revealed that PACAP38 infusion induced an increase in facial blood flow, heart rate and mild headache, and indicated that these PACAP38-induced responses were inhibited by Lu AG09222.

**Conclusions:**

This proof-of-mechanism study demonstrated that Lu AG09222 inhibited PACAP38-induced cephalic vasodilation and increases in heart rate, and reduced concomitant headache. Lu AG09222 may be a potential therapy against migraine and other PACAP-mediated diseases.

**Trial registration:**

ClinicalTrials.gov: NCT04976309. Registration date: July 19, 2021.

**Graphical Abstract:**

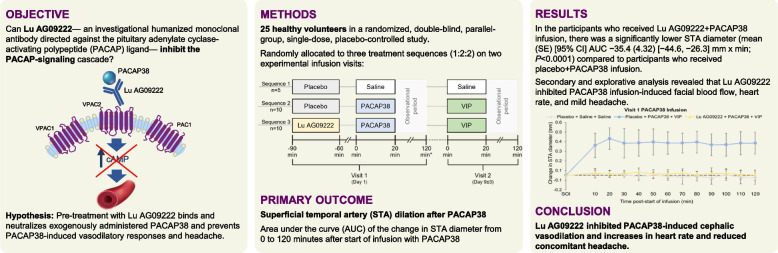

**Supplementary Information:**

The online version contains supplementary material available at 10.1186/s10194-023-01599-w.

## Background

Migraine is a common neurological disease affecting 1 billion people worldwide and the leading cause of disability in individuals younger than 50 years of age [1]. Advances in research have led to the discovery of molecular pathways involved in migraine and the development of mechanism-based therapies [2].

Pituitary adenylate cyclase-activating polypeptide (PACAP) and vasoactive intestinal peptide (VIP) are part of the same glucagon/secretin superfamily of structurally related vasoactive peptides and share significant similarities [3]. They exert their effects through three distinct common G-protein‒coupled receptors: VIP receptor 1 (VPAC1), VIP receptor 2 (VPAC2) and PACAP type 1 (PAC1) receptor [4, 5]. PACAP and the common receptors are expressed in the trigeminovascular system, which is the proposed anatomical and neurophysiological substrate for migraine [6]. Amongst various biological effects, PACAP stimulates an increase of intracellular second messenger cyclic adenosine monophosphate (cAMP) and downstream signal, causing vasodilation [7, 8]. PACAP exists in two isoforms: PACAP38 and PACAP27 [9, 10]. In humans, PACAP38 dilates extracerebral arteries, induces headache in healthy volunteers and induces migraine attacks in individuals with migraine [11–14]. Elevated plasma levels of PACAP38 have been reported during migraine attacks—both compared interictally in patients with migraine and in the overall population of patients with migraine [15]. Collectively, these data support an important role of PACAP in migraine pathophysiology and its potential as a novel drug target [16].

An investigational humanized monoclonal antibody directed against PACAP, Lu AG09222, is currently being developed for migraine prevention [17]. Lu AG09222 binds to PACAP and inhibits receptor binding [17, 18]. Whether Lu AG09222 can prevent physiological responses of PACAP38 is unknown.

This proof-of-mechanism randomized controlled trial investigated the effect of Lu AG09222 on vascular responses and headache after PACAP38 and VIP infusion in healthy volunteers. We hypothesized that pre-treatment with Lu AG09222 binds and neutralizes exogenously administered PACAP38 and prevents PACAP38-induced vasodilatory responses and headache, thereby confirming target engagement in this human model [19, 20]. Since VIP interacts with the same receptors as PACAP [4, 5], we also explored whether pre-treatment with Lu AG09222 affected VIP-induced vasodilatory responses.

## Materials and methods

### Study design and participants

This was a phase 1, interventional, randomized, double-blind, parallel-group, placebo-controlled, single-dose study investigating the effect of Lu AG09222 in a headache model with healthy volunteers. This study was designed in accordance with the Declaration of Helsinki and conducted in compliance with Good Clinical Practice and applicable regulatory requirements; all participants provided informed consent prior to participation. There were four notable protocol deviations, none of which affected the integrity of the study or subject safety; see Additional file 1, which provides supplementary methods. The study was conducted between 15 July 2021 and 10 December 2021 at the Danish Headache Center in Copenhagen, Denmark, and is registered with ClinicalTrials.gov (NCT04976309).

Healthy adults aged 18–45 years (inclusive) with a body mass index ≥ 18.0 and ≤ 30.0 kg/m^2^, a body weight ≥ 45 and ≤ 95 kg and vitals as specified in Additional file 1 at the screening visit were able to participate in this study. Individuals were excluded if they fulfilled the diagnostic criteria for a primary headache disorder, except infrequent tension-type headache (≤ 1 day per month on average for 6 months prior to inclusion), had a first-degree relative with a primary headache disorder, according to the International Classification of Headache Disorders, 3^rd^ edition (ICHD-3) [21], had any clinically significant medical, neurological or psychiatric disease, or other major disorders. Full selection criteria can be found in Additional file 1, with a summary of essential lifestyle restrictions and protocol deviations in Additional file 1.

### Randomization, masking, and interventions

**Fig. 1:**
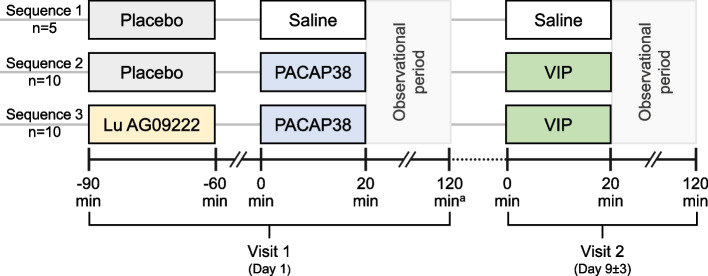
Study design. ^a^Key endpoints assessed at 120 min are: change in STA (primary outcome) and RA diameter (measured by high-resolution ultrasonography), change in heart rate, and change in facial blood flow (measured by speckle contrast imager). PACAP38, pituitary adenylate cyclase-activating polypeptide 38; RA, radial artery; STA, superficial temporal artery; VIP, vasoactive intestinal peptide

Potential participants were assessed for eligibility and screened (Additional file 1). Twenty-five study participants were randomized manually via a sponsor-generated manual randomization list (1:2:2; stratified by sex) into three treatment sequences: (1) placebo (0.9% isotonic saline, single-dose intravenous infusion over 30 min) + saline (intravenous infusion; visit 1) + saline (intravenous infusion visit 2); (2) placebo (0.9% isotonic saline, single-dose intravenous infusion over 30 min) + PACAP38 (10 pmol/kg/min; visit 1) + VIP (8 pmol/kg/min; visit 2); and (3) Lu AG09222 (single-dose intravenous infusion over 30 min at a dose calculated to bind all endogenous PACAP and exogenous infused PACAP38 [data on file]) + PACAP38 (10 pmol/kg/min; visit 1) + VIP (8 pmol/kg/min; visit 2). Trained personnel at the clinical site were responsible for preparing Lu AG09222 or placebo, and PACAP38, VIP and saline IV infusions in a double-blind fashion (blinded to the investigator and subjects). The personnel responsible for receiving, storing, preparing and dispensing Lu AG09222, PACAP38, VIP and saline were unblinded and were not responsible for other aspects of the clinical study where blinding was necessary. The infusion bags administered to the participants were identical in appearance and labelled by the site after dose preparation by the unblinded personnel in a manner that protected blinding. The study treatments (Lu AG09222 or placebo) were administered 90 min before start of the PACAP38 or saline infusion on visit 1. Twenty-minute infusions of PACAP38 or saline (visit 1) and VIP or saline (visit 2) were followed by an observation period of 100 min, during which the participants remained resting in a supine position (until timepoint 120 min). All participants attended a safety follow-up visit 10 to 12 weeks after study drug administration (Fig. 1).

### Data collection and outcome measures

Vascular parameters (superficial temporal artery [STA] diameter and radial artery [RA] diameter measured by high-resolution ultrasonography; facial flushing measured by speckle contrast imager), heart rate and headache were documented before Lu AG09222 or placebo infusion and PACAP38 or saline infusion at visit 1 and before VIP or saline infusion at visit 2. After PACAP38, VIP and saline infusions, vascular parameters, heart rate and headache were documented every 10 min from 0 to 2 h. After the observation period, participants were provided with a headache diary and discharged from the clinic. The diary was filled out every hour from 3 to 8 h (or until sleep) after the start of infusion with PACAP38, VIP, or saline, then at 24 h and daily thereafter until 1 week after the VIP or second saline infusion (if applicable). Participants recorded the presence of a headache and evaluated headache intensity, characteristics and localization, facial flushing, other associated symptoms and use of acute medication.

The primary outcome measure was the area under the curve (AUC) of the change in STA diameter from 0 to 120 min after start of PACAP38 infusion (AUC_STA-PACAP38_). Secondary outcome measures assessed changes in vasodilation, facial blood flow, heart rate, safety and tolerability. Exploratory outcomes included headache occurrence and intensity. A summary of the study objectives and endpoints can be found in Additional file 1. Information on the dose of Lu AG09222 and exploratory biomarker endpoints have been omitted from the manuscript due to being confidential at this point.

### Statistical analysis

A sample size of 10 participants per treatment sequence provided more than 90% power to detect a difference in the AUC of change from the start of infusion of PACAP38 in STA diameter (AUC_STA-PACAP38_; primary outcome measure) of 34 mm × min, with a standard deviation of 14 mm × min in the placebo + PACAP38 + VIP treatment sequence and a standard deviation of 4 mm × min in the Lu AG09222 + PACAP38 + VIP treatment sequence, at a two-sided 5% significance level. All testing was performed based on a 5% two-sided significance level.

All randomized participants who received either placebo or Lu AG09222 were included in the safety analysis; all participants who received an infusion of PACAP38 or saline and had a valid pre-infusion STA diameter measurement and a valid AUC_STA-PACAP38_ measurement on visit 1 were included in analyses related to the PACAP38 infusion; and all participants who received a VIP or saline infusion and had a valid pre-infusion STA diameter measurement and a valid AUC_STA-VIP_ measurement on visit 2 were included in analyses related to the VIP infusion.

The primary endpoint was analyzed using an analysis of covariance (i.e., change from start of infusion in AUC_STA-PACAP38_), using treatment sequence and sex as factors and the last pre-infusion STA measurement as a covariate. Only for the primary outcome for the comparison of Lu AG09222 + PACAP38 versus placebo + PACAP38 was the type I error formally controlled; all other analyses were considered exploratory and significance was considered indicative rather than confirmative for the finding. All confidence intervals (CIs) are 95% unless otherwise specified. Methods for handling missing data can be found in Additional file 1. Descriptive statistics are presented for continuous variables as well as the categorical variables counts and percentages (if applicable). Data analyses were performed using the SAS® statistical software package Version 9.4 or higher.

### Data availability

In accordance with EFPIA’s and PhRMA’s ‘Principles for Responsible Clinical Trial Data Sharing’ guidelines, Lundbeck is committed to responsible sharing of clinical trial data in a manner that is consistent with safeguarding the privacy of patients, respecting the integrity of national regulatory systems and protecting the intellectual property of the sponsor. The protection of intellectual property ensures continued research and innovation in the pharmaceutical industry. Deidentified data are available to those whose request has been reviewed and approved through an application submitted to https://www.lundbeck.com/global/our-science/clinical-data-sharing.

## Results

A total of 25 adults with a mean (standard deviation [SD]) age of 27 (7.7) years were randomized (placebo + saline + saline, *n* = 5; placebo + PACAP38 + VIP, *n* = 10; Lu AG09222 + PACAP38 + VIP, *n* = 10). Participants were predominantly White (18/25 [72.0%]) and female (15/25 [60.0%]), with a mean (SD) body mass index of 22.7 (3.09) kg/m^2^. Baseline demographic and clinical characteristics were similar across treatment groups (Table 1).

**Table 1:** Baseline demographic and clinical characteristics

	**Placebo + saline + saline** ***n* = 5**	**Placebo + PACAP38 + VIP** ***n* = 10**	**Lu AG09222 + PACAP38 + VIP** ***n* = 10**	**Overall** ***n* = 25**
Age, years, mean (SD)	30 (8.5)	26 (7.6)	27 (8.0)	27 (7.7)
Sex, n (%)				
Male	2 (40)	4 (40)	4 (40)	10 (40)
Female	3 (60)	6 (60)	6 (60)	15 (60)
Race, n (%)				
Asian	1 (20)	1 (10)	1 (10)	3 (12)
White	4 (80)	6 (60)	8 (80)	18 (72)
Other	0	3 (30)	1 (10)	4 (16)
Body mass index, kg/m^2^, mean (SD)	22.0 (3.75)	22.3 (2.50)	23.5 (3.44)	22.7 (3.09)
Superficial temporal artery diameter, mm, mean (SD)				
Visit 1	1.12 (0.165)	1.02 (0.174)	1.08 (0.229)	1.06 (0.192)
Visit 2	1.04 (0.284)	0.92 (0.128)	1.07 (0.217)	1.00 (0.205)
Radial artery diameter, mm, mean (SD)				
Visit 1	2.34 (0.464)	2.12 (0.340)	2.41 (0.470)	2.28 (0.425)
Visit 2	2.31 (0.381)	2.10 (0.288)	2.34 (0.398)	2.24 (0.358)
Facial blood flow, flux, mean (SD)				
Visit 1	539 (52.7)	570 (111)	527 (155)	547 (120)
Visit 2	509 (95.7)	519 (104)	587 (153)	544 (125)
Heart rate, bpm, mean (SD)				
Visit 1	58.6 (4.16)	64.0 (11.6)	59.8 (8.78)	61.2 (9.38)
Visit 2	59.0 (8.63)	57.4 (8.53)	59.3 (9.56)	58.5 (8.65)

*Bpm* Beats per minute, *PACAP38* Pituitary adenylate cyclase-activating polypeptide 38, *SD* Standard deviation, *VIP* Vasoactive intestinal peptide

**Fig. 2:**
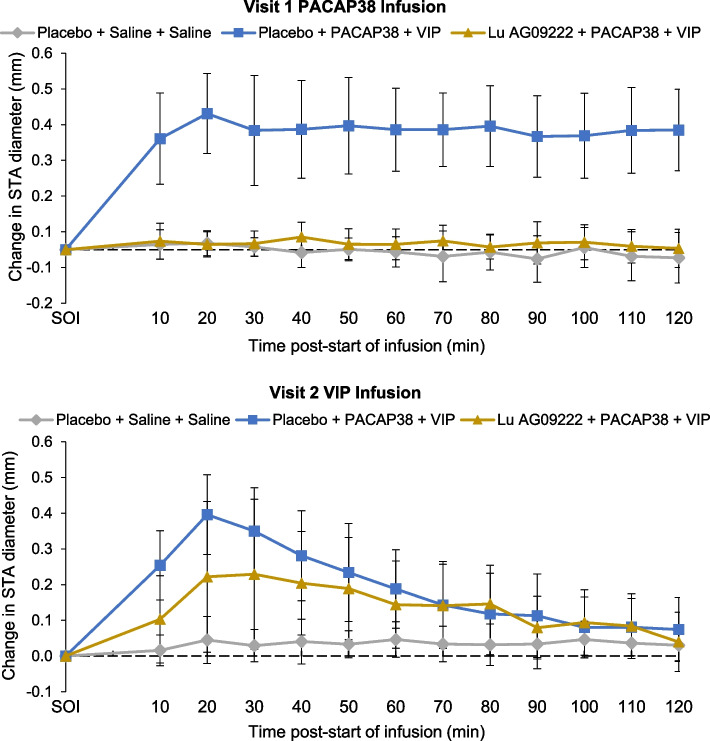
Mean change in STA diameter from start of PACAP38/VIP infusion. PACAP38, pituitary adenylate cyclase-activating polypeptide 38; STA, superficial temporal artery; VIP, vasoactive intestinal peptide

**Fig. 3:**
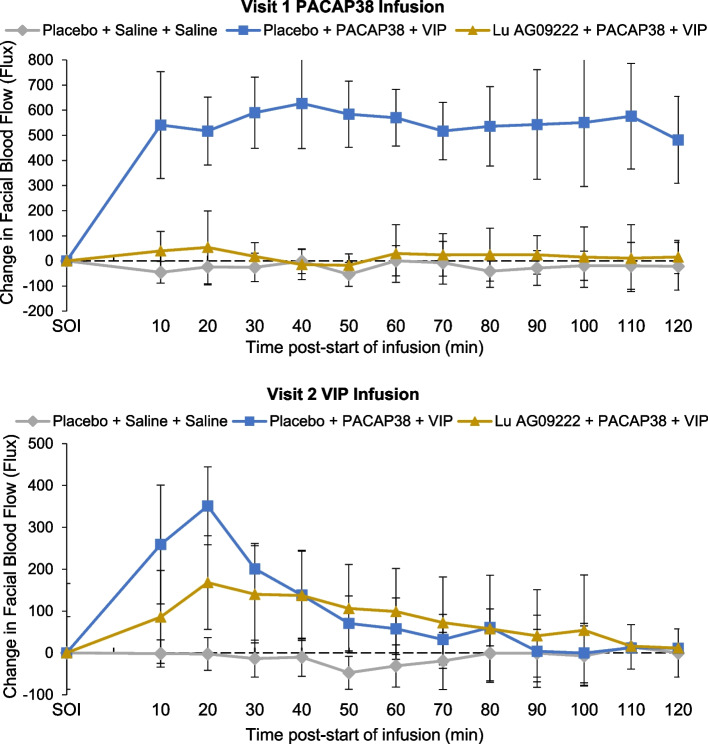
Mean change in facial blood flow from start of PACAP38/VIP infusion. PACAP38, pituitary adenylate cyclase-activating polypeptide 38; VIP, vasoactive intestinal peptide

**Fig. 4:**
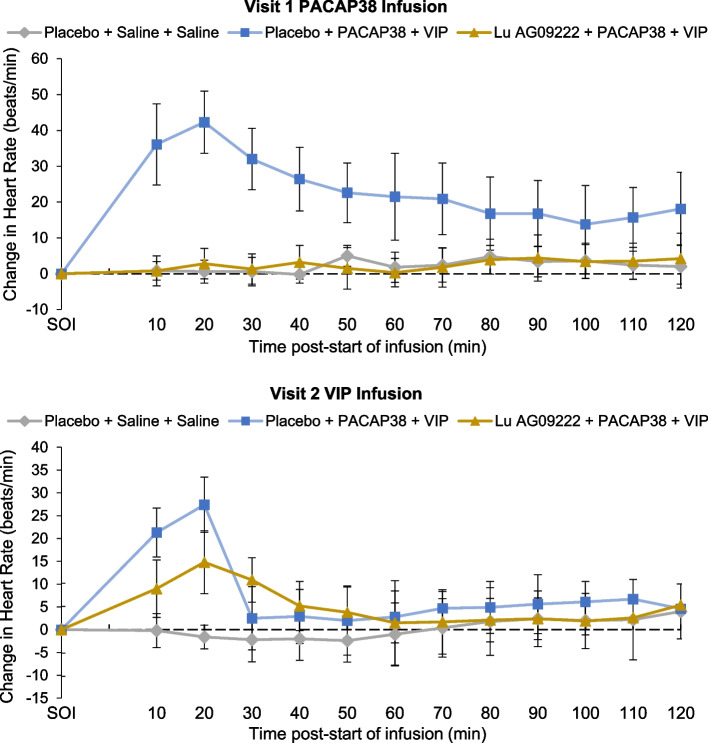
Mean change in heart rate from start of PACAP38/VIP infusion. PACAP38, pituitary adenylate cyclase-activating polypeptide 38; VIP, vasoactive intestinal peptide

In participants who received placebo before PACAP38 infusion, there was a significantly greater STA diameter (mean (SE) [95% CI]; 37.3 (6.46) [23.1, 51.5] mm × min; *P* = 0.0001) in mean AUC_STA-PACAP38_, compared with participants who received placebo before saline, confirming that PACAP38 induced STA vasodilation in the absence of Lu AG09222 (Table 2). In participants who received Lu AG09222 before PACAP38 infusion, there was a significantly lower mean STA diameter (mean (SE) [95% CI]; ‒35.4 (4.32) [‒44.6, ‒26.3] mm × min; *P* < 0.0001) in mean AUC_STA-PACAP38_ compared to participants who received placebo before PACAP38 infusion (Table 2, Fig. 2). Lu AG09222 also prevented PACAP38-induced increases in facial blood flow (Table 2, Fig. 3) and heart rate (Table 2, Fig. 4). Lu AG09222 did not prevent VIP-induced dilation of the STA, facial blood flow AUC or heart rate increase. There was a significant difference in maximum facial blood flow after VIP infusion between placebo- and Lu AG09222-treated groups (Table 3, Fig. 3). No significant increase was observed in the RA diameter after PACAP38 or VIP infusion compared to saline (Tables 2 and 3). The effects of Lu AG09222 on PACAP38- and VIP-induced vasodilation, facial flushing, heart rate and headache intensity are summarized in Tables 2 and 3, respectively.

**Table 2:** Effects of Lu AG09222 following PACAP38 infusion^a^

	**Placebo + saline + saline** ***n* = 5**	**Placebo + PACAP38 + VIP** ***n* = 10**	**Lu AG09222 + PACAP38 + VIP** ***n* = 10**
**Superficial temporal artery diameter**			
AUC_STA-PACAP38_, mm × min, mean (SD)	‒0.49 (5.03)	38.6 (13.0)	2.01 (3.96)
LS mean difference (SE) [95% CI]		37.3 (6.46) [23.1, 51.5]	‒35.4 (4.32) [‒44.6, ‒26.3]
*P*-value		0.0001^b^	< 0.0001^c^
CFI_STA-60 min-PACAP38_, mm, mean (SD)	‒0.006 (0.043)	0.336 (0.116)	0.015 (0.043)
LS mean difference (SE) [95% CI]			‒0.313 (0.041) [‒0.399, ‒0.227]
*P*-value			< 0.0001^c^
max_STA-PACAP38_, mm, mean (SD)	0.046 (0.026)	0.410 (0.112)	0.060 (0.036)
LS mean difference (SE) [95% CI]			‒0.340 (0.036) [‒0.416, ‒0.264]
*P*-value			< 0.0001^c^
**Radial artery diameter**			
AUC_RA-PACAP38_, mm × min, mean (SD)	2.79 (7.41)	2.01 (6.78)	6.26 (10.5)
LS mean difference (SE) [95% CI]		‒3.82 (3.27) [‒11.0, 3.38]	3.12 (4.67) [‒6.79, 13.0]
*P*-value		0.2676^b^	
CFI_RA-60 min-PACAP38_, mm, mean (SD)	0.021 (0.066)	0.047 (0.102)	0.056 (0.123)
LS mean difference (SE) [95% CI]			0.0003 (0.060) [‒0.127, 0.127]
max_RA-PACAP38_, mm, mean (SD)	0.062 (0.066)	0.098 (0.102)	0.108 (0.112)
LS mean difference (SE) [95% CI]			‒0.013 (0.054) [‒0.127, 0.101]
**Facial blood flow**			
AUC_FBF-PACAP38_, flux × min, mean (SD)	‒2755 (6572)	63,907 (16,516)	2113 (8197)
LS mean difference (SE) [95% CI]		64,321 (7158) [48,567, 80,075]	‒60,200 (5808) [‒72,500, ‒47,900]
*P*-value		< 0.0001^b^	< 0.0001^c^
max_FBF-PACAP38_, flux, mean (SD)	36.6 (46.2)	665 (190)	126 (162)
LS mean difference (SE) [95% CI]			‒505 (72.1) [‒658, ‒352]
*P*-value			< 0.0001^c^
**Heart rate**			
AUC_HR-PACAP38_, beats, mean (SD)	262 (282)	2792 (1001)	290 (337)
LS mean difference (SE) [95% CI]			‒2520 (359) [‒3280, ‒1750]
*P*-value			< 0.0001^c^
**Headache intensity**			
AUC_HI-PACAP38 0-8 h_, points, mean (SD)	169 (204)	762 (725)	156 (324)
LS mean difference (SE) [95% CI]			‒606 (252) [‒1140, ‒74.1]
* P*-value			0.0279^c^
max_HI-PACAP38_, points, mean (SD)	2.00 (1.87)	2.60 (2.07)	1.00 (1.76)
LS mean difference (SE) [95% CI]			‒1.60 (0.775) [‒3.23, 0.034]
*P*-value			0.0544^c^

*AUC* Area under the curve, *AUC*_*FBF-PACAP38*_ AUC in change in facial blood flow from 0 to 120 min after start of infusion (SOI refers to start of infusion of PACAP38, VIP, or saline), *AUC*_*HI-PACAP38 0-8 h*_ AUC for headache intensity from 0 to 8 h after SOI, *AUC*_*HR-PACAP38*_ AUC in change in heart rate from 0 to 120 min after SOI, *AUC*_*RA-PACAP38*_ AUC in change in RA diameter from 0 to 120 min after SOI, *AUC*_*STA-PACAP38*_ AUC of change in STA diameter from 0 to 120 min after SOI, *CFI* Change from SOI, *CFI*_*RA-60 min-PACAP38*_ Change in RA diameter from 0 to 60 min after SOI, *CFI*_*STA-60 min-PACAP38*_ Change in STA diameter from 0 to 60 min after SOI, *CI* Confidence interval, *HR* Heart rate, *LS* Least squares, *max*_*FBF-PACAP38*_ Maximum change in facial blood flow between 0 and 120 min after SOI; *max*_*HI-PACAP38*_ Peak headache score between 0 and 24 h after SOI, *max*_*RA-PACAP38*_ Maximum change in RA diameter between 0 and 120 min after SOI; *max*_*STA-PACAP38*_ Maximum change in STA diameter between 0 and 120 min after SOI, *PACAP38* Pituitary adenylate cyclase-activating polypeptide 38, *RA* Radial artery, *SD* Standard deviation, *SE* Standard error, *STA* Superficial temporal artery, *VIP* Vasoactive intestinal peptide

^a^Group-specific estimates are presented as mean (SD); all differences are expressed as LS mean differences (SE); note that least-squares mean differences may slightly differ from observed mean differences due to the adjustment for the baseline value

^b^*P*-value vs. placebo + saline + saline was calculated for AUC values only (if no significant difference was detected between the two control groups [placebo + saline + saline and placebo + PACAP38 + VIP], then no further analysis was conducted to compare Lu AG09222 + PACAP38 + VIP and placebo + PACAP38 + VIP)

^c^*P*-value vs. placebo + PACAP38 + VIP

**Table 3:** Effects of Lu AG09222 following VIP infusion^a^

	**Placebo + saline + saline** ***n* = 5**	**Placebo + PACAP38 + VIP** ***n* = 10**	**Lu AG09222 + PACAP38 + VIP** ***n* = 10**
**Superficial temporal artery diameter**			
AUC_STA-VIP_, mm × min, mean (SD)	4.06 (5.47)	22.7 (11.4)	16.5 (12.4)
LS mean difference (SE) [95% CI]		18.1 (6.10) [4.68, 31.5]	‒6.49 (6.82) [‒20.9, 7.97]
*P*-value		0.0128^b^	0.3555^c^
CFI_STA-60 min-VIP_, mm, mean (SD)	0.047 (0.050)	0.188 (0.110)	0.144 (0.122)
LS mean difference (SE) [95% CI]			‒0.045 (0.068) [‒0.188, 0.099]
*P*-value			0.5173^c^
max_STA-VIP_, mm, mean (SD)	0.071 (0.051)	0.405 (0.107)	0.274 (0.208)
LS mean difference (SE) [95% CI]			‒0.124 (0.091) [‒0.316, 0.068]
*P*-value			0.1894^c^
**Radial artery diameter**			
AUC_RA-VIP_, mm × min, mean (SD)	‒1.09 (4.34)	1.23 (6.49)	9.31 (6.24)
LS mean difference (SE) [95% CI]		1.16 (3.69) [‒6.95, 9.28]	11.6 (2.83) [5.59, 17.6]
*P*-value		0.7578^b^	
CFI_RA-60 min-VIP_, mm, mean (SD)	0.006 (0.045)	0.017 (0.066)	0.075 (0.052)
LS mean difference (SE) [95% CI]			0.077 (0.030) [0.013, 0.141]
max_RA-VIP_, mm, mean (SD)	0.034 (0.23)	0.087 (0.097)	0.135 (0.062)
LS mean difference (SE) [95% CI]			0.0773 (0.0403) [‒0.00811, 0.163]
**Facial blood flow**			
AUC_FBF-VIP_, flux × min, mean (SD)	‒1216 (4420)	11,919 (6959)	9827 (10,983)
LS mean difference (SE) [95% CI]		12,865 (3129) [5977, 19,752]	‒3210 (4337) [‒12,400, 5985]
*P*-value		0.0017^b^	0.4701^c^
max_FBF-VIP_, flux, mean (SD)	55.4 (31.0)	366 (169)	199 (99.8)
LS mean difference (SE) [95% CI]			–214 (53.6) [–328, –100]
*P*-value			0.0011^c^
**Heart rate**			
AUC_HR-VIP_, beats, mean (SD)	14.8 (524)	897 (560)	585 (568)
LS mean difference (SE) [95% CI]		853 (295) [203, 1502]	–271 (215) [–728, 186]
*P*-value		0.0147^b^	0.2265^c^
**Headache intensity**			
AUC_HI-VIP 0-8 h_, points, mean (SD)	35.9 (80.3)	104 (178)	88.4 (179)
LS mean difference (SE) [95% CI]			-15.2 (82.2) [–189, 158]
*P*-value			0.8554^c^
max_HI-VIP_, points, mean (SD)	0.400 (0.894)	1.10 (0.994)	0.800 (1.87)
LS mean difference (SE) [95% CI]			-0.300 (0.690) [–1.76, 1.16]
*P*-value			0.6692^c^

*AUC* Area under the curve, *AUC*_*FBF-VIP*_ AUC in change in facial blood flow from 0 to 120 min after start of infusion (SOI refers to start of infusion of PACAP38, VIP, or saline), *AUC*_*HI-VIP 0-8 h*_ AUC for headache intensity from 0 to 8 h after SOI, *AUC*_*HR-VIP*_ AUC in change in heart rate from 0 to 120 min after SOI, *AUC*_*RA-VIP*_ AUC in change in RA diameter from 0 to 120 min after SOI, *AUC*_*STA-VIP*_ AUC of change in STA diameter from 0 to 120 min after SOI, *CFI* Change from SOI, *CFI*_*RA-60 min-VIP*_ Change in RA diameter from 0 to 60 min after SOI, *CFI*_*STA-60 min-VIP*_ Change in STA diameter from 0 to 60 min after SOI, *CI* Confidence interval, *HR* Heart rate, *LS* Least squares, *max*_*FBF-VIP*_ Maximum change in facial blood flow between 0 and 120 min after SOI, *max*_*HI-VIP*_ Peak headache score between 0 and 24 h after SOI, *max*_*RA-VIP*_ Maximum change in RA diameter between 0 and 120 min after SOI, *max*_*STA-VIP*_ Maximum change in STA diameter between 0 and 120 min after SOI, *PACAP38* Pituitary adenylate cyclase-activating polypeptide 38, *RA* Radial artery, *SD* Standard deviation, *SE* Standard error, *STA* Superficial temporal artery, *VIP* Vasoactive intestinal peptide

^a^Group-specific estimates are presented as mean (SD); all differences are expressed as least-squares mean differences (SE); note that least-squares mean differences may slightly differ from observed mean differences due to the adjustment for the baseline value

^b^*P*-value vs. placebo + saline + saline was calculated for AUC values only (if no significant difference was detected between the two control groups [placebo + saline + saline and placebo + PACAP38 + VIP], then no further analysis was conducted to compare Lu AG09222 + PACAP38 + VIP and placebo + PACAP38 + VIP)

^c^*P*-value vs. placebo + PACAP38 + VIP

AUC for headache intensity after 8 h following PACAP38 infusion (AUC_HI-PACAP38 0-8 h_) was significantly lower in participants who received Lu AG09222 before PACAP38 infusion compared to participants who received placebo before PACAP38 infusion (AUC_HI-PACAP38 0-8 h_ mean [SE]: 169 [204] versus 762 [725], *P* = 0.0279, respectively). There was no significant difference in peak headache score up to 24 h after PACAP38 infusion in the two groups (max_HI-PACAP38_, *P* = 0.0544, Table 2).

Treatment-emergent adverse events divided into three periods are summarized in Table 4. No serious adverse events were reported. Review of the safety labs, vital signs, electrocardiograms, weight/body mass index and Columbia–Suicide Severity Rating Scale scores indicated no safety trends or concerns. Additionally, no participants were positive for anti-drug antibodies after receiving Lu AG09222.

**Table 4:** Treatment-emergent adverse events in ≥ 2 subjects by system organ class

	**Placebo + saline + saline** ***n* = 5**	**Placebo + PACAP38 + VIP** ***n* = 10**	**Lu AG09222 + PACAP38 + VIP** ***n* = 10**
**Period 1**^**a**^			
Feeling hot	2 (40.0)	0	0
Headache	1 (20.0)	0	0
**Period 2**^**b**^			
Headache	4 (80.0)	9 (90.0)	4 (40.0)
Feeling hot	0	10 (100)	3 (30.0)
Flushing	0	10 (100)	1 (10.0)
Palpitations	0	10 (100)	0
Fatigue	2 (40.0)	2 (20.0)	2 (20.0)
Musculoskeletal stiffness	0	3 (30.0)	1 (10.0)
Disturbance in attention	1 (20.0)	0	2 (20.0)
Nasal congestion	1 (20.0)	2 (20.0)	0
Photosensitivity reaction	1 (20.0)	2 (20.0)	0
Vomiting	0	2 (20.0)	0
**Period 3**^**c**^			
Headache	2 (40.0)	7 (70.0)	4 (40.0)
Feeling hot	0	10 (100)	3 (30.0)
Flushing	0	9 (90.0)	4 (40.0)
Palpitations	0	6 (60.0)	0
Fatigue	0	1 (10.0)	2 (20.0)
Musculoskeletal stiffness	0	0	1 (10.0)
Disturbance in attention	0	1 (10.0)	1 (10.0)
Photosensitivity reaction	0	1 (10.0)	0

*AE* Adverse event, *PACAP38* Pituitary adenylate cyclase-activating polypeptide 38, *VIP* Vasoactive intestinal peptide

^a^Period 1: AEs that started after dosing of the study drug (Lu AG09222 or placebo) but before PACAP38 or saline infusion

^b^Period 2: AEs that started during or after the PACAP38 or saline infusion at visit 1, but before VIP or saline infusion at visit 2

^c^Period 3: AEs that started during or after the VIP or saline infusion at visit 2

## Discussion

Mechanism-specific migraine preventive therapy can reduce frequency and severity of migraine attacks and improve migraine-related disability [22]. Experimental models of migraine have mapped cellular mechanisms of migraine pathophysiology related to specific molecular agents [19, 20]. These models have been integral to the development of migraine-specific preventive therapy. Results from a recent study applying a mouse model of migraine support that PACAP acts via an independent pathway, and therefore presents a potential novel target for preventive migraine therapy [23]. In the present proof-of-mechanism randomized controlled trial, we used the well-established experimental model of migraine/headache with PACAP38 [19], known to induce dilation of extracerebral arteries, increased heart rate, facial flushing and headache in healthy volunteers. Here, Lu AG09222 prevented these PACAP38-induced physiological responses.

Endogenous PACAP plays a role in the regulation of cephalic haemodynamics and is present in perivascular parasympathetic and trigeminal afferent fibers [24–26]. PACAP receptors (VPAC1, VPAC2 and PAC1) [4, 5] are expressed in the trigeminovascular system, and activation causes an intracellular increase in cAMP that can result in vasodilation, among other effects [7, 8, 27]. The role of PACAP and its vasodilatory properties related to migraine are supported by previous findings showing that intravenous infusion of PACAP38 dilates extracerebral arteries [11–14], as well as induces headache in healthy volunteers (100%) [12, 13] and migraine attacks in people with migraine (58–73%) [12, 14]. The model has been validated by studies testing the effect of anti-migraine treatment on PACAP38-induced vasodilation, headache and migraine attacks. The anti-migraine drug sumatriptan reduced PACAP38-induced changes of STA and middle meningeal artery circumference and prevented headache in healthy volunteers when administered before PACAP38 infusion [13, 28]. In a randomized controlled trial, migraine patients treated early with sumatriptan developed fewer migraine attacks after PACAP38 infusion (15%) compared to patients receiving placebo (42%) [29].

The PAC1 receptor has previously been suggested as a potential target for treating migraine, inhibiting a key part of the PACAP signalling pathway, since PACAP38 has high affinity on this receptor [16]. In patients with migraine, a proof-of-concept study using a PAC1 receptor monoclonal antibody did not meet its primary endpoint of migraine prevention [30], suggesting that blocking the PAC1 receptor alone was not effective for preventing migraine attacks. As an alternative, targeting the PACAP ligand could be a better strategy since PACAP perhaps exerts its migraine-inducing effects through VPAC1 or VPAC2, or a combination of receptors [30].

Lu AG09222—which in this study prevents PACAP38-induced vasodilatory responses and headache, thereby confirming target engagement—is not dependent on receptors but instead binds and neutralizes the PACAP ligand; therefore, by inhibiting PACAP from binding to its target receptors (PAC1, VPAC1 and VPAC2), the PACAP signalling cascade may be prevented from initiating any physiological responses [17]. The current study demonstrates the ability of Lu AG09222 to block dilation of extracerebral arteries mediated by exogenous PACAP38, and the adverse event frequency was lower in the Lu AG09222-treated group. We propose that Lu AG09222 would also inhibit a physiological vascular response mediated by endogenous PACAP, as the exogenous PACAP38, applied in the provocation model, results in higher plasma concentrations than endogenous PACAP [31]. This is supported by findings in the previous report of the pharmacological characterization of ALD1910 (i.e. Lu AG09222) [17], reporting that it inhibited endogenously released PACAP in an animal model of neurogenic vasodilation and parasympathetic lacrimation. The present study demonstrates a preventive effect on PACAP38-induced headache of Lu AG09222, as headache intensity and duration measured after PACAP38 infusion were lower in participants who received Lu AG09222 compared to participants who received placebo before PACAP38 infusion. The current findings provide support for the potential for Lu AG09222 in migraine prevention. A randomized, double-blind, proof-of-concept phase 2a trial was recently completed assessing efficacy, safety, and tolerability of Lu AG09222 in the prevention of migraine (NCT05133323). At the time of submission for this manuscript, the trial is under analysis and in reporting phase.

VIP infusion induced a short-lived vasodilation of STA, facial flushing and heart rate increase, compared to saline, in the placebo group. This is consistent with previous findings of VIP-induced cephalic vasodilation in healthy volunteers [32] and substantiates the physiological response induced by VIP infusion. There was no difference in the AUC of STA diameter, facial blood flow AUC and heart rate between placebo and Lu AG09222 after VIP infusion, but point estimates at 20 min post-infusion hint at partial blocking and there was a significant difference in maximum flushing. Furthermore, the adverse event frequency was lower in the Lu AG09222-treated group. In a previous study, 2-h infusion with VIP induced migraine attacks in patients with migraine at an induction rate of 71% [33], similar to results reported in a separate study after 20-min PACAP38 infusion [14], suggesting a potential yet much less pronounced role for VIP in migraine pathogenesis that remains to be explored in future studies. Variability and lack of power hinders further interpretation. To the best of our knowledge, no other studies have evaluated how blocking PACAP ligand affects VIP-induced vascular response. Exploring the interrelationship between PACAP and VIP would be valuable to elucidate a possible link between PACAP and VIP beyond sharing common receptors.

### Strengths and limitations

This study has a relatively small sample size. However, the study was adequately powered based on the study design and considering previous findings of substantial STA dilation in provocation studies with PACAP38 [12, 29]. The study design involved a set order of infusion visits: first infusion visit with PACAP38 and second infusion visit with VIP. Based on the Lu AG09222 half-life in rats (approximately 8 days) [17] and estimated half-life in humans from the first-in-human clinical trial (data on file), Lu AG09222 should still be sufficiently present during the second infusion visit. The differential effect of Lu AG09222 on PACAP38 and VIP infusion visits could have also been studied, for example, if the order of PACAP38 and VIP infusions had been randomized or by including a second group of participants who would have received VIP on the first visit and PACAP38 on the second visit. A previous study measured PACAP38 mean plasma half-life as 3.5 ± 1.3 min [11]. The 6-day minimum requirement between experimental visits was included to avoid carry-over effect of PACAP38 to second infusion visit. Adverse events, or lack thereof (e.g., facial flushing, heart palpitations) from PACAP38 and VIP infusions may, to some degree, have compromised blinding both for the study participants and investigators. This was, in view of the known physiological response of PACAP38 and VIP infusion in healthy volunteers, taken into consideration in study design by including a placebo + saline + saline group that mimics the hypothesized blocking response after Lu AG09222 treatment. Participants were young (mean 27 years) and predominantly female, corresponding well with the phenotypical characteristics of migraine patients, though generalizability may be limited.

## Conclusion

Lu AG09222 significantly inhibited PACAP38-induced cephalic vasodilation and reduced concomitant headache in healthy volunteers. These results demonstrate that Lu AG09222 binds to and effectively inhibits PACAP38-mediated physiological responses and indicate Lu AG09222 as a future treatment for migraine and other conditions that would benefit from inhibition of the PACAP signalling cascade.

## Supplementary Information

**Additional file 1: Supplementary Methods.** Extended inclusion criteria: lifestyle restrictions summary. Handling of missing data. Protocol deviations. Investigators. **Supplementary Table 1.** Full selection criteria. **Supplementary Table 2.** Study objectives and endpoints. **Supplementary Figure 1.** Enrollment and study flowchart.

## Data Availability

The dataset supporting the conclusions of this article is are available to those whose request has been reviewed and approved through an application submitted to https://www.lundbeck.com/global/our-science/clinical-data-sharing.

## Abbreviations

AUC: Area under the curve
cAMP: Cyclic adenosine monophosphate
PACAP: Pituitary adenylate cyclase-activating polypeptide
PAC1: Pituitary adenylate cyclase-activating polypeptide type I receptor
RA: Radial artery
STA: Superficial temporal artery
VIP: Vasoactive intestinal peptide
VPAC1/2: Vasoactive intestinal peptide receptor type 1/2
