# Correction: The effect of Lu AG09222 on PACAP38- and VIP-induced vasodilation, heart rate increase, and headache in healthy subjects: an interventional, randomized, double-blind, parallel-group, placebo-controlled study

**DOI:** 10.1186/s10194-023-01610-4

**Published:** 2023-06-19

**Authors:** Nadja Bredo Rasmussen, Christina Deligianni, Casper Emil Christensen, William Kristian Karlsson, Haidar Muhsen Al-Khazali, Tom Van de Casteele, Charlotte Granhall, Faisal Mohammad Amin, Messoud Ashina

**Affiliations:** 1 Department of Neurology, Danish Headache Center, Rigshospitalet Glostrup, Faculty of Health and Medical Sciences, University of Copenhagen, Valdemar Hansen Vej 5, 2600 Glostrup, Denmark; 2 Department of Clinical Medicine, Faculty of Health and Medical Sciences, University of Copenhagen, Valdemar Hansen Vej 5, 2600 Glostrup, Denmark; 3 H. Lundbeck A/S, Ottiliavej 9, 2500 Valby, Denmark; 4 Department of Neurorehabilitation/Traumatic Brain Injury, Rigshospitalet Glostrup Faculty of Health and Medical Sciences, University of Copenhagen, Valdemar Hansen Vej 5, 2600 Glostrup, Denmark

**Correction: J Headache Pain 24, 60 (2023)**

**https://doi.org/10.1186/s10194-023-01599-w**

In this article [1] the graphical abstract was missing and should have appeared as below:


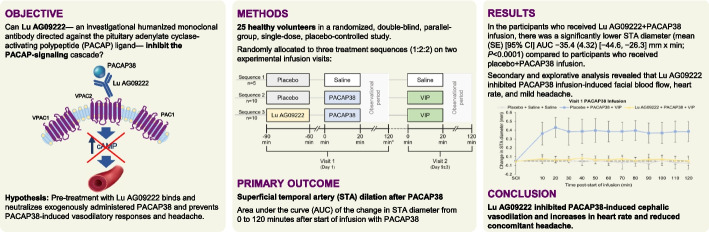


## References

[1] Rasmussen NB, Deligianni C, Christensen CE (2023). The effect of Lu AG09222 on PACAP38- and VIP-induced vasodilation, heart rate increase, and headache in healthy subjects: an interventional, randomized, double-blind, parallel-group, placebo-controlled study. J Headache Pain.

