# Supplementary material for: The effect of Lu AG09222 on PACAP38- and VIP-induced vasodilation, heart rate increase, and headache in healthy subjects: an interventional, randomized, double-blind, parallel-group, placebo-controlled study
Source: J Headache Pain. 2023 May 25;24(1):60. doi: 10.1186/s10194-023-01599-w (PMC10210362; doi:10.1186/s10194-023-01599-w)
Supplement: Supplementary file 1 — Additional file 1: Supplementary Methods. Extended inclusion criteria: lifestyle restrictions summary. Handling of missing data. Protocol deviations. Investigators. Supplementary Table 1. Full selection criteria. Supplementary Table 2. Study objectives and endpoints. Supplementary Figure 1. Enrollment and study flowchart. [file 10194_2023_1599_MOESM1_ESM.pdf]

Supplemental material for

PACAP-targeted antibody Lu AG09222 inhibits vasodilation in healthy subjects

Nadja Bredo Rasmussen,[1] Christina Deligianni,[1] Casper Emil Christensen,[1] William Kristian Karlsson,[1] Haidar Muhsen Al-Khazali,[1] Tom Van de Casteele,[2] Charlotte Granhall,[2] Faisal Mohammad Amin,[1,3] and Messoud Ashina[1,4]

[1]Department of Neurology, Danish Headache Center, Copenhagen University Hospital - Rigshospitalet, Copenhagen, Denmark; [2]H. Lundbeck A/S, Copenhagen, Denmark; [3]Department of Neurorehabilitation/Traumatic Brain Injury, Rigshospitalet, University of Copenhagen, Copenhagen, Denmark; [4]Department of Clinical Medicine, University of Copenhagen, Copenhagen, Denmark

Contents

Supplementary Methods

Supplementary Table 1 Full selection criteria

Supplementary Table 2 Study objectives and endpoints

Supplementary Figure 1 Enrollment and study flowchart

Supplementary Methods

Extended inclusion criteria: lifestyle restrictions summary

The total study duration per participant from randomization to the follow-up visit was 10–12 weeks. During the study duration, participants had to adhere to the following lifestyle restrictions: refrain from COVID-19 vaccination within 7 days before the screening visit and until 9 days after study drug administration; and before the days of PACAP38 and VIP infusion: abstain from use of any over-the-counter, herbal or prescribed medication (except contraception) within 48 hours (or 5 half-lives, whichever is shorter); abstain from consuming coffee, tea, cola, cocoa and other caffeine-containing beverages/foods within 12 hours; abstain from alcohol, drugs, smoking and use of tobacco/nicotine products within 12 hours; fast for at least 4 hours before the visits; and must not have had any headache within 24 hours before infusion or any clinically significant illness between visit 1 and visit 2.

Handling of missing data

For re-assessments of safety variables (clinical safety laboratory tests, vital signs, ECGs), the following principle will be applied for missing data: the data point closest in time to the planned assessment will be used in the summary tables, provided that this data point is considered valid and pertains to the relevant time period. For safety data and efficacy data, if the time/date of the SOI is incomplete or missing, it will be excluded from the calculation unless the incomplete date/time indicates the value was recorded prior to PACAP38, VIP or saline dosing at that challenge visit. Area under the curve (AUC) values will be derived following the linear trapezoidal rule using actual times. Nominal times may be used if actual times are missing. An AUC will not be calculated if there are fewer than eight post-dose results available for each infusion visit or if three consecutive post-dose results are missing. The last pre-infusion value will be used for timepoint 0. For headache intensity score, if a participant is missing a timepoint due to being asleep, the last observation carried forward (LOCF) approach will be used to impute these timepoints. This rule will be utilized for parameter calculation, summaries and analysis of the headache intensity score data.

For the adverse event data, the following rules will apply:

- For the derivation of causality (applicable to adverse events captured on the 'Adverse event intensity log' eCRF form only): The causality to study treatment is not captured

on the adverse event intensity log electronic case report form (eCRF) because it is always assumed to be the same as the causality captured on the first eCRF completed for this adverse event (initial intensity). Therefore, for these cases the missing causality will be set to that captured on the first form completed for this adverse event.

- For the derivation of treatment-emergent status (applicable to all adverse events): If the start date/time of an adverse event is incomplete or missing, an adverse event will be assumed to not be a treatment-emergent adverse event (TEAE), unless the incomplete start date/time or the end date/time indicates an adverse event started after study drug dosing.
- For the derivation of TEAEs causally related to the use of Lu AG09222, PACAP38, VIP, placebo or saline (applicable to TEAEs only): If the study treatment causality for a TEAE is missing, a TEAE will be assumed to be a causally related TEAE.
- For the derivation of onset time / time relative to dose of Lu AG09222, PACAP38, VIP, placebo or saline (applicable to TEAEs only): If the start date/time of a TEAE is missing, onset time will not be calculated. If the start date/time of a TEAE is incomplete, where possible, the possible onset time will be calculated and presented as '<X days'. The same rules will be followed for the non-study drug. Any clock changes will be accounted for in the derivation.
- For the derivation of duration (applicable to all adverse events): If the end date/time of an adverse event is missing, duration will not be calculated. If the start or end date/time of an adverse event is incomplete, where possible, the duration will be calculated and presented as '<X days'. Any clock changes will be accounted for in the derivation.
- For the calculation of TEAE summary statistics: If the intensity of a TEAE is missing, it will not be imputed. If an intensity of a TEAE changes, the TEAE will be summarized under the maximum intensity recorded.

Protocol deviations

The important protocol deviations that occurred during the study are:

- For three subjects, haematology results at screening were not provided by the central laboratory due to courier issue on late delivery and blood clotting. For all three subjects, the blood samples were repeated at Visit 1 and analyzed at the local laboratory, in addition to the collection of the protocol-specified blood sample. The haematology values obtained from the local laboratory were reviewed before IMP administration.

For all the subjects, the results were within reference ranges and subject eligibility was thereby confirmed.

- For one subject, the STA diameter was not performed on Day 1 as per protocol at 50 minutes and 110 minutes due to an ongoing adverse event of abdominal pain.

None of these deviations from the protocol affected the integrity of the study or subject safety. There were no protocol deviations related to COVID-19 during the study.

Investigators

Primary Investigator (PI):	Messoud Ashina, MD, PhD, DMSc
Sub-Investigators:	Nadja Bredo Rasmussen, MD, PhD fellow
	Christina Deligianni, MD, FEBN
	William Kristian Karlsson, MD, PhD fellow
	Thien Phu Do, MD, PhD
	Casper Emil Christensen, MD, PhD
	Faisal Mohammad Amin, MD, PhD

Supplementary Table 1 Full selection criteria

Inclusion Criteria
Subjects are eligible to be included in the study only if all the following criteria apply:

1. The subject is able to read and understand the *Subject Information Sheet* and *Informed Consent Form*.
2. The subject has signed the study-specific *Informed Consent Form.*
3. The subject is a man or woman.
4. The subject is ≥18 and ≤45 years of age at the screening visit.
5. The subject has a body mass index (BMI) ≥18.0 and ≤30.0 kg/m^2, and a body weight ≥45 and ≤95 kg at the screening visit.
6. The subject, if a woman, must:
    a. remain sexually abstinent, when this is in line with her preferred and usual lifestyle OR
    b. engage exclusively in same-sex relationships OR
    c. agree to avoid becoming pregnant from the screening visit until 6 months after the last dose of the study drug, AND
    d. use a highly effective contraceptive method as required by local regulation or practice if she is of childbearing potential and she has a male partner. The contraceptive method must be used from visit 1 until 6 months after the last dose of the study drug and must include one of the following: intrauterine device; contraception implant; progesterone-only pills; injectable progestogen (Depo-Provera®); combination hormonal contraceptive method (tablets, patches, or vaginal ring with both oestrogen and progestogen), OR
    e. have had her last natural menstruation ≥12 months prior to the screening visit (confirmed by FSH level), OR
    f. have had a hysterectomy prior to the screening visit, OR
    g. have been surgically sterilized prior to the screening visit, OR
    h. have a male partner who was surgically sterilized prior to the screening visit AND
    i. not donate ova until 6 months after the last dose of the study drug
7. The subject, if a man, must:
    a. remain sexually abstinent, when this is in line with his preferred and usual lifestyle OR
    b. engage exclusively in same-sex relationships OR
    c. agree to avoid impregnating his partner from the screening visit until 6 months after the last dose of the study drug, AND
    d. use a highly effective contraceptive method as required by local regulation or practice if his female partner is of childbearing potential. The contraceptive method must be used from visit 1 until 6 months after the last dose of the study drug and must include one of the following: intrauterine device; contraception implant; progesterone-only pills; injectable progestogen (Depo-Provera®); combination hormonal contraceptive method (tablets, patches, or vaginal ring with both estrogen and progestogen), OR
    e. have been surgically sterilized prior to the screening visit, OR
    f. have a partner who had her last natural menstruation ≥12 months prior to the screening visit, OR
    g. have a partner who had a hysterectomy prior to the screening visit, OR
    h. have a partner who was surgically sterilized prior to the screening visit AND
    i. not donate sperm until ≥6 months after the last dose of the study drug
8. The subject has a resting supine pulse ≥50 and ≤100 bpm at the screening visit. For physically/athletically well-trained subjects, the lower limit is ≥45 bpm.
9. The subject has a resting supine systolic blood pressure ≥90 and ≤150 mmHg and a resting supine diastolic blood pressure ≥50 and ≤90 mmHg at the screening visit.
10. The subject is, in the opinion of the investigator, generally healthy based on medical history, a physical examination, vital signs, an ECG, and the results of the clinical chemistry, haematology, urinalysis, serology and other laboratory tests at screening.

Exclusion Criteria
Subjects are excluded from the study if any of the following criteria apply:

1. The subject fulfills the diagnostic criteria for a primary headache disorder or has a first-degree relative with a primary headache disorder, according to the International Headache Society (IHS) *International Classification of Headache Disorders* 3rd edition (ICHD-3), except tension-type headache.
2. The subject has or has had tension-type headache more than once per month on average during the 6 months prior to the screening visit.
3. The subject is pregnant or breastfeeding.
4. The subject has taken disallowed medication <5 half-lives prior to the screening visit for any medication taken. Disallowed medication is any prescribed medication or over-the counter medication as well as any herbal medicine known to interfere with the metabolic CYP pathways, such as St. John's Wort, ginseng, milk thistle and echinacea. Subjects who have taken any non-prescribed systemic or topical medication may participate in the study if, in the opinion of the investigator, the medication will not interfere with the study procedures, study results, or compromise safety. Use of contraceptives, routine vitamins and occasional use of over-the-counter analgesics, proton pump inhibitors and asthma/allergy medication (on demand) is allowed.
5. The subject has orthostatic hypotension, defined as a decrease in systolic blood pressure ≥20 mmHg from supine to standing, at the screening visit.
6. The subject has a QTc interval >430 ms as the cut-off at the screening visit, as calculated by the ECG equipment and evaluated by the investigator. The ECG may be repeated if any of the values are out-of-range or abnormal.
7. The subject has or has had any clinically significant immunological, cardiovascular, respiratory, metabolic, renal, hepatic, gastrointestinal, endocrinological, haematological, dermatological, venereal, neurological, or psychiatric disease or other major disorder.
8. The subject has a history of cancer, other than basal cell or Stage 1 squamous cell carcinoma of the skin or adequately treated cervical intraepithelial neoplasia, that has not been in remission for >5 years prior to the first dose of the study drug.
9. The subject has had major surgery (excluding laparoscopic cholecystectomy or uncomplicated appendectomy) <6 months prior to the first dose of the study drug.
10. The subject has had a clinically significant illness <4 weeks prior to the screening visit.

11. The subject has one or more clinical laboratory test values outside the reference range, based on the blood and urine taken at the screening visit. Borderline values, except for liver test values (bilirubin, ALP, ALT, AST) above the upper limit of the reference range, may be accepted if they are, in the opinion of the investigator, clinically insignificant.
12. The subject has tested positive for HIV, HBsAg or anti-HCV.
13. The subject has had significant blood loss <3 months prior to the first dose of the study drug.
14. The subject has donated blood <3 months prior to the first dose of the study drug.
15. The subject has significant alcohol consumption, defined as an alcohol intake >21 units per week for men or >14 units per week for women. A unit of alcohol is defined as 250 mL of lager/beer, 100 mL of wine, or 25 mL of spirits.
16. The subject has tested positive at the screening visit for drugs of abuse.
17. The subject has taken any investigational medicinal product <5 half-lives prior to the first dose of the study drug.
18. The subject has a history of severe drug allergy or hypersensitivity.
19. The subject is, in the opinion of the investigator, unlikely to comply with the protocol or is unsuitable for any reason.
20. The subject has received COVID-19 vaccine <7 days prior to the screening visit.
21. The subject is at significant risk of suicide (defined, using the C-SSRS, as the subject answering: 'yes' to suicidal ideation questions 4 or 5 or answering: 'yes' to suicidal behavior within the past 12 months.

ALP denotes alkaline phosphatase; ALT alanine aminotransferase; AST aspartate transaminase; anti-HCV anti-hepatitis C virus; BMI body mass index; bpm beats per minute; CSSR Columbia-Suicide Severity Rating Scale; CYP cytochrome P450; ECG electrocardiogram; FSH follicle-stimulating hormone; HBsAg hepatitis B surface antigen; HIV human immunodeficiency virus; IHS ICHD-3 International Headache Society International Classification of Headache Disorders 3rd edition; mmHg millimeters of mercury; ms millisecond; QTc heart-rate corrected QT interval.

Supplementary Table 2 Study objectives and endpoints

Objectives	Endpoints
**Primary**	
To investigate the effect of Lu AG09222 on vasodilation after SOI of PACAP38	AUC of change from SOI in STA diameter from 0 to 120 min after SOI of PACAP38 ($\text{AUC}_{\text{STA-PACAP38}}$)
**Secondary**	
1. To investigate the effect of Lu AG09222 on vasodilation after SOI of VIP<br><br>2. To investigate the effect of Lu AG09222 on facial flushing (i.e. facial blood flow) after SOI of PACAP38 and VIP<br><br>3. To investigate the effect of Lu AG09222 on heart rate after SOI of PACAP38 and VIP<br><br>4. To investigate safety and tolerability of Lu AG09222	Vasodilation<br>• Change from SOI in STA diameter to 60 min after SOI of PACAP38 ($\text{CFI}_{\text{STA-60min-PACAP38}}$)<br>• Maximum change from SOI in STA diameter between 0 and 120 min after SOI of PACAP38 ($\text{max}_{\text{STA-PACAP38}}$)<br>• AUC in change from SOI in STA diameter from 0 to 120 min after SOI of VIP ($\text{AUC}_{\text{STA-VIP}}$)<br>• Change from SOI in STA diameter from SOI to 60 min of VIP ($\text{CFI}_{\text{STA-60min-VIP}}$)<br>• Maximum change from SOI in STA diameter between 0 and 120 min after SOI of VIP ($\text{max}_{\text{STA-VIP}}$)<br>• AUC in change from SOI in RA diameter from 0 to 120 min after SOI of PACAP38 or VIP ($\text{AUC}_{\text{RA-PACAP38}}$, $\text{AUC}_{\text{RA-VIP}}$)<br>• Change from SOI in RA diameter to 60 min after SOI of PACAP38 or VIP (CFIRA-60min-PACAP38, $\text{CFI}_{\text{RA-60min-VIP}}$)<br>• Maximum change from SOI in RA diameter between 0 and 120 min after SOI of PACAP38 or VIP ($\text{max}_{\text{RA-PACAP38}}$, $\text{max}_{\text{RA-VIP}}$)<br><br>Facial blood flow<br>• AUC in change from SOI in facial blood flow from 0 to 120 min after SOI of PACAP38 or VIP ($\text{AUC}_{\text{FBF-PACAP38}}$, $\text{AUC}_{\text{FBF-VIP}}$)<br>• Maximum change from SOI in facial blood flow between 0 and 120 min after SOI of PACAP38 or VIP ($\text{max}_{\text{FBF-PACAP38}}$, $\text{max}_{\text{FBF-VIP}}$)<br><br>Heart rate<br>• AUC in change from SOI in heart rate from 0 to 120 min after SOI of PACAP38 or VIP ($\text{AUC}_{\text{HR-PACAP38}}$, $\text{AUC}_{\text{HR-VIP}}$)<br><br>Safety and tolerability<br>• Adverse events<br>• Absolute values and changes from baseline in clinical safety laboratory test values, vital signs<br>• ECG assessments<br>• Potentially clinically significant clinical safety laboratory test values, vital signs<br>• C-SSRS score<br>• Presence of anti-drug antibodies (ADAs)<br>• Characterization of ADA-positive samples for neutralizing antibodies
**Exploratory**[a]	
1. To explore the effect of Lu AG09222 on headache intensity<br><br>2. To determine the relationship, if any, between Lu AG09222 serum levels (PK) and PD data	Headache<br>• Occurrence of headache (headache intensity score >0) between SOI and 24 hours after SOI of PACAP38 or VIP<br>• AUC for headache intensity from 0 to 8 hours after SOI of PACAP38 or VIP ($\text{AUC}_{\text{HI-PACAP38 0-8h}}$, $\text{AUC}_{\text{HI-VIP 0-8h}}$)<br>• Peak headache score between SOI and 24 hours after SOI of PACAP38 or VIP ($\text{max}_{\text{HI-PACAP38}}$, $\text{max}_{\text{HI-VIP}}$)<br>• Use of acute medication between SOI and 24 hours after SOI of PACAP38 or VIP<br><br>Pharmacokinetics<br>• Serum concentrations of Lu AG09222

[a]Some exploratory endpoints have been omitted from the manuscript due to being confidential information at this point.

AUC denotes area under the curve; CFI change from start of infusion; C-SSRS Columbia–Suicide Severity Rating Scale; ECG electrocardiogram; PACAP38 pituitary adenylate cyclase-activating polypeptide 38; PD pharmacodynamic; PK pharmacokinetic; RA radial artery; SOI start of infusion; STA superficial temporal artery; VIP vasoactive intestinal peptide.

Supplementary Figure 1. Enrollment and study flowchart

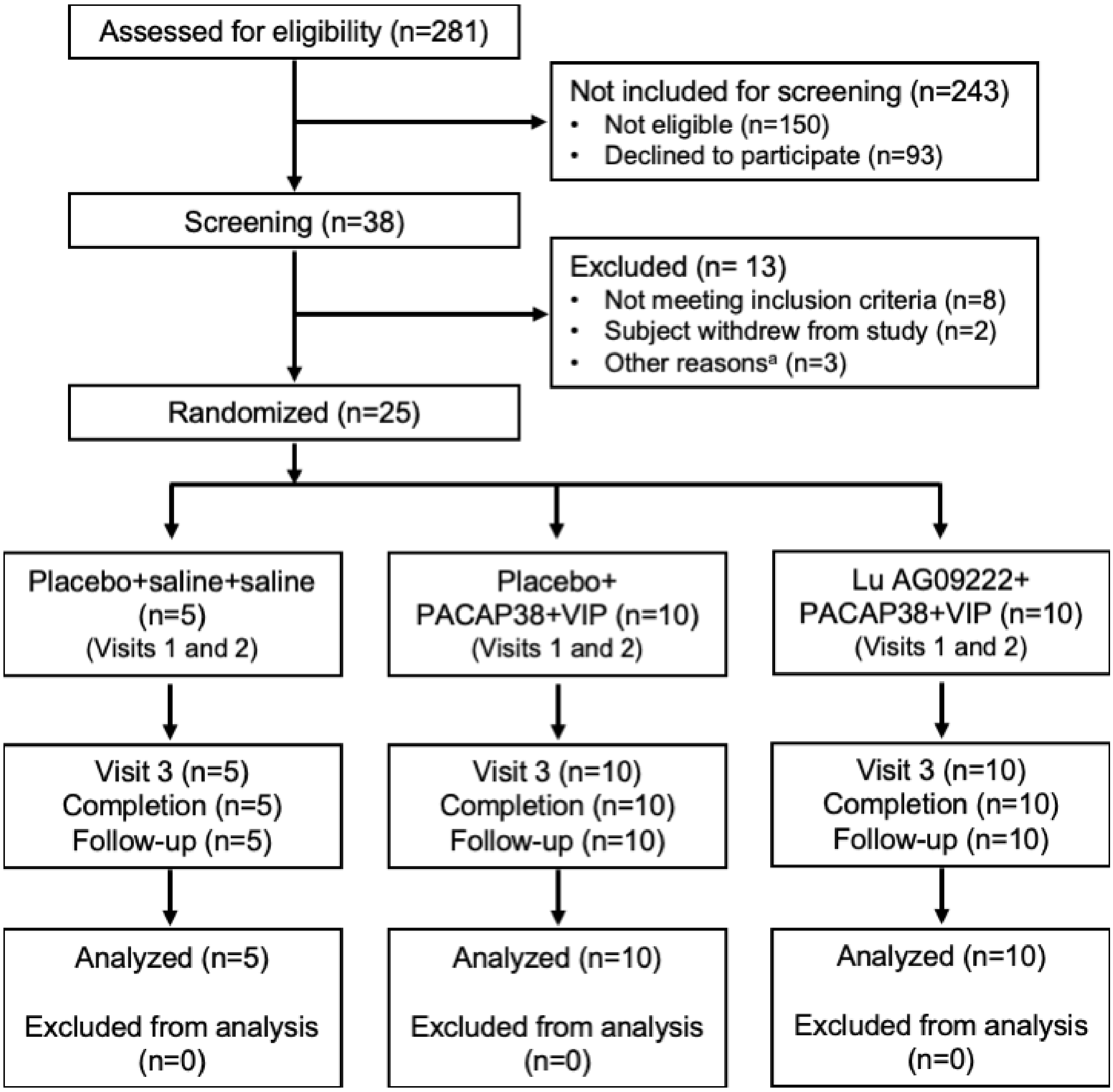

Healthy volunteers were screened and randomized according to the flow diagram shown above.

[a]Delayed due to unsuitable blood samples: n=2. Randomization for the study completed: n=1.

Visit 3 was utilized for blood sample collection to test for anti-drug antibodies (ADAs), PACAP and Lu AG09222 quantification, and evaluate adverse events and recent and concomitant medication.

PACAP38 denotes pituitary adenylate cyclase-activating polypeptide 38; VIP vasoactive intestinal polypeptide.
